# Enteric Glia at the Crossroads between Intestinal Immune System and Epithelial Barrier: Implications for Parkinson Disease

**DOI:** 10.3390/ijms21239199

**Published:** 2020-12-02

**Authors:** Laura Benvenuti, Vanessa D’Antongiovanni, Carolina Pellegrini, Luca Antonioli, Nunzia Bernardini, Corrado Blandizzi, Matteo Fornai

**Affiliations:** 1 Department of Clinical and Experimental Medicine, University of Pisa, 56126 Pisa, Italy; laura.benvenuti962@gmail.com (L.B.); v.dantongiovanni@gmail.com (V.D.); nunzia.bernardini@med.unipi.it (N.B.); corrado.blandizzi@med.unipi.it (C.B.); matteo.fornai@unipi.it (M.F.); 2 Department of Pharmacy, University of Pisa, 56126 Pisa, Italy; carolina.pellegrini@unipi.it

**Keywords:** enteric glial cells, Parkinson’s disease, intestinal epithelial barrier, gut–brain axis, enteric immune system, α-synuclein

## Abstract

Over recent years, several investigations have suggested that Parkinson’s disease (PD) can be regarded as the consequence of a bowel disorder. Indeed, gastrointestinal symptoms can occur at all stages of this neurodegenerative disease and in up to a third of cases, their onset can precede the involvement of the central nervous system. Recent data suggest that enteric glial cells (EGCs) may play a major role in PD-related gastrointestinal disturbances, as well as in the development and progression of the central disease. In addition to their trophic and structural functions, EGCs are crucial for the homeostatic control of a wide range of gastrointestinal activities. The main purpose of this review was to provide a detailed overview of the role of EGCs in intestinal PD-associated alterations, with particular regard for their participation in digestive and central inflammation as well as the dynamic interactions between glial cells and intestinal epithelial barrier. Accumulating evidence suggests that several pathological intestinal conditions, associated with an impairment of barrier permeability, may trigger dysfunctions of EGCs and their shift towards a proinflammatory phenotype. The reactive gliosis is likely responsible for PD-related neuroinflammation and the associated pathological changes in the ENS. Thus, ameliorating the efficiency of mucosal barrier, as well as avoiding IEB disruption and the related reactive gliosis, might theoretically prevent the onset of PD or, at least, counteract its progression.

## 1. Introduction

Parkinson’s disease (PD) is one of the most common neurodegenerative disorders in the world, with an incidence rising almost exponentially with age. This has significant public health implications, since it is estimated that the number of people with PD will grow significantly by 2030 as a consequence of increased life expectancy [1].

PD is characterized by a significant dopaminergic neuronal loss in the nigrostriatal pathway, which is responsible for the occurrence of typical motor symptoms [2,3]. In addition, PD patients are characterized by the occurrence of gastrointestinal (GI) motor dysfunctions, which are classically considered as secondary conditions comprised in the wide spectrum of non-motor symptoms associated with PD. However, over recent years, several lines of evidence have lent support to the hypothesis that PD could be considered a gut-originated disease [4]. Indeed, the onset of GI symptoms can precede the involvement of central nervous system (CNS) in up to 30% of PD patients, and bowel dysfunctions are among the most severe non-motor symptoms of the disease [5]. As a consequence, the hypothesis of a digestive origin of PD has taken shape and paved the way to several studies focused on the evaluation of bowel alterations associated with PD [6,7,8,9].

Accumulating evidence, yielded by studies of the gut to unravel PD pre-symptomatic progression, sustains a very intriguing theory on the gut alterations that may anticipate and predict the onset of PD symptoms, and may help to identify innovative anti-PD therapeutic interventions. In this setting, it has been proposed that enteric glial cells (EGCs) could take a significant part in PD-related GI dysfunctions, as well as in the induction and progression of the CNS disease. In this review, we discuss recent research data pointing out the involvement of enteric glia in the pathophysiology of bowel alterations associated with PD, as a possible starting point for the development of new therapeutic strategies targeting these cells.

## 2. Methods

PubMed was used as the database to establish and identify the literature. No date restriction was put on the search, even though articles from the last five years were preferred. The literature search was carried out using different combinations of keywords: “Parkinson Disease” AND (Gut OR EGC OR IEB OR α-syn). Articles selected from PubMed were reviewed first by title, then abstract, and finally, the full article.

## 3. The Enteric Glial Cells

The enteric nervous system (ENS) includes a large population of EGCs, which are thought to be the digestive equivalent of brain astrocytes [10]. EGCs are historically considered to contribute mainly to the structural and trophic protection of enteric neurons [11]. However, in addition to these traditional tasks, EGCs have been found to play crucial roles in the homeostatic control of a broad variety of GI functions, including motility, synaptic transmission, mucosal barrier integrity, neurogenesis, and neuroprotection, via dynamic interplays with neurons, epithelial, and immune cells [12,13].

In mice, similarly for enteric neurons, EGCs are generated from proliferating neural crest-derived cells that colonize the embryonic intestine from day 9 to 13.5 [14,15]. Shortly after their birth, EGCs migrate from the myenteric plexus and submucous plexus into the lamina propria to colonize the mucosal layer [16].

**Figure 1:**
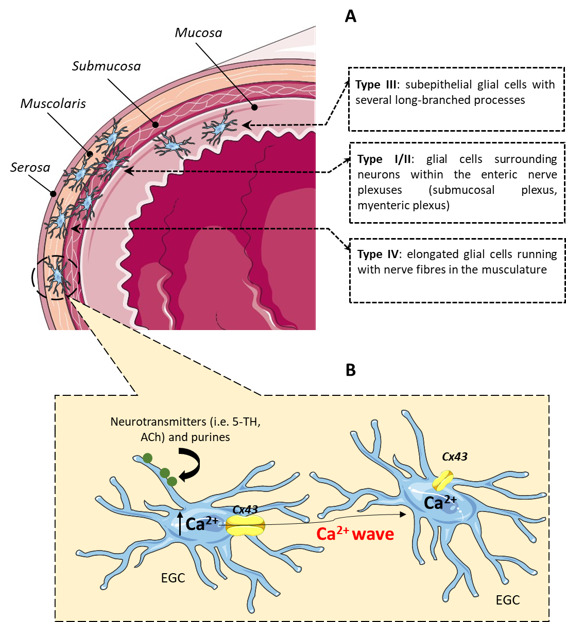
Schematic representation of the distribution and activation of EGCs. (**A**) Type III glial cells are located directly beneath the epithelial cells; type I/II glial cells surround neurons within the enteric nerve plexuses (submucosal plexus and myenteric plexus); type IV glial cells running with nerve fibers in the musculature. (**B**) Neurotransmitters or purines, released from enteric neurons, can activate EGCs, triggering an increase in intracellular Ca^2+^ concentration with consequent opening of Cx43 hemichannels. EGCs use Ca^2+^ signaling to communicate with each other and transmit information to distant sites within the ENS. Abbreviations: Ca^2+^, calcium; Cx43, connexin-43; EGC, enteric glial cell; ENS, enteric nervous system; 5-HT, serotonin; ACh, acetylcholine.

Typically, EGCs can be classified into four subgroups, based on their structure and location within the digestive tract [12]. Thus, type I or “protoplasmic” glial cells are star-shaped cells located in the ganglia; type II or “fibrous” cells are found in the interganglionic connectives, lining the nerves; type III or “mucosal” and type IV or “intramuscular” EGCs are located in their respective site within the gut wall [12] (Figure 1).

Along with differences in location and morphology, mature EGCs express a variety of molecular markers, such as glial fibrillary acidic protein (GFAP) [17], S100β [18], connexin-43 (Cx43) [19], proteolipid protein 1 (PLP1) [20], and vimentin [17]. These markers are not pan-expressed, as the expression of GFAP in the adult stage is fleeting, but Sox10 and PLP1 label the large majority of EGCs. These complex patterns of protein expression are likely to reflect the functional diversity and plasticity of different EGC types [21].

Enteric glial cell populations and their respective morpho-functional properties often differ significantly according to sex, age, and species [12,19,22]. This is a critical aspect when comparing observations from different studies and interpolating data from animal and human studies. For instance, in the rodent (guinea pig) intestine, the glia-to-neuron ratio is approximately seven-fold less than in the human digestive tract [22]. Furthermore, EGCs may display variability in the expression of specific receptor subtypes among different species or gut regions [23,24,25]. Many signal pathways appear well maintained, but how close human and murine enteric glia really are remains unclear.

EGCs play a significant part in the modulation of gut functions by regulating motility and influencing both neural networks and intestinal epithelium [23,25,26]. Like astrocytes, EGCs are excitable cells and their excitability is mediated mainly by cytosolic calcium (Ca^2+^) signals [25,27], triggered by neurotransmitters, such as serotonin or acetylcholine, through synaptic interactions [21,25] (Figure 1). McClain and colleagues showed that Ca^2+^ signals in EGCs regulate the opening of glial Cx43 hemichannels, and that EGC-specific ablation of the Cx43 gene decreases intestinal smooth muscle contractions under both ex vivo and in vivo recording [19]. Glial Ca^2+^ dynamics are extremely important for normal intestinal physiology and in any circumstance, modifications that affect EGCs’ signaling pathways, such as variations in their number or Ca^2+^ signaling, could play a major role in GI motor disorders [19,23].

## 4. Role of Enteric Glia in Intestinal and CNS Inflammation

Over the past years, several studies have shown that the pathophysiology of GI diseases, such as inflammatory bowel diseases and chronic constipation, can significantly involve EGCs [10,28,29].

**Figure 2:**
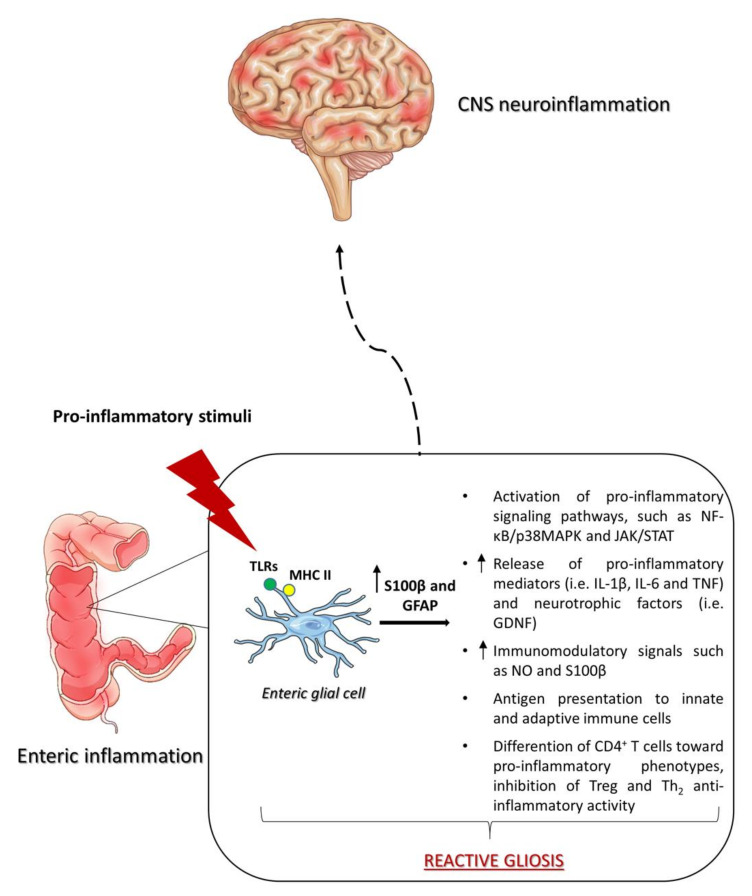
Role of EGCs in intestinal and CNS inflammation. In the bowel, pro-inflammatory stimuli, including IL-1, IL-6, and LPS elicit a pathological activation of the enteric glia, known as “reactive gliosis”. As a consequence, the expression of glial markers, such as GFAP and S100β, increase significantly, triggering the release of pro-inflammatory cytokines (i.e., TNF, IL-1β, and IL-6), glial cell-derived neurotrophic factors (i.e., GDNF), and other immunomodulatory signaling molecules, including NO and S100β. In addition, the activated EGCs act as antigen-presenting cells at the neuron–epithelial interface, triggering an abnormal activation of CD4+ T cells. This cascade of events amplifies the pro-inflammatory environment, intestinal barrier damage, and neurochemical changes leading to a neuroinflammatory response that, somehow, ascends to the CNS. Abbreviations: CNS, central nervous system; EGC, enteric glial cell; GDNF, glial cell-derived neurotrophic factor; GFAP, glial fibrillary acidic protein; IL, interleukin; LPS, lipopolysaccharide; MHC II, major histocompatibility complex II; NO, nitric oxide; TLRs, toll-like receptors; TNF, tumor necrosis factor.

A number of investigations have shown that EGCs express the major histocompatibility complex II (MHC II) and can respond to harmful inputs, mostly through toll-like receptors (TLR)-2 and -4, thus actively controlling the neuro–immune axis and protecting the host against gut pathogens [11] (Figure 2).

This glial response under pathological conditions, known as “reactive gliosis”, usually triggers a broad spectrum of alterations, including TLRs activation [30,31], altered expression and release of several glia-related proteins and enzymes, such as GFAP, S100B, inducible nitric oxide synthase (iNOS), and matrix metallopeptidases [32], and the activation of pro-inflammatory signaling pathways, such as NFκB/p38MAPK and JAK/STAT [33]. Activated EGCs display an altered sensitivity to purinergic agonists and respond to cell threats through the release of cytokines, such as tumor necrosis factor (TNF), interleukin (IL)-1β and IL-6, growth factors, including GDNF, and other immunomodulatory signals, such as nitric oxide (NO) and S100B [25,34,35], all contributing to the development of pro-inflammatory conditions, which could negatively affect the intestinal barrier integrity and activate neuronal death [26] (Figure 2). Therefore, it appears that the enteric glia is actively involved in intestinal inflammatory responses, as it is now clear that EGCs hold the ability of modulating immune functions, secreting and responding directly to inflammatory mediators [36].

As anticipated above, EGCs react to the exposure to harmful stimuli-expressing TLRs and other co-stimulatory T cell molecules, including MHC-II [37]. It is not clear whether these signaling mechanisms are involved in other facets of reactive gliosis, outside of the inflamed bowel. However, as the most prevalent cell population in the ENS, EGCs are the most likely candidates to operate as antigen-presenting cells in the intestinal neuroimmune system. Indeed, they are capable of presenting antigens to both innate and adaptive immune cells located in the digestive tract [38] (Figure 2). Peripheral inflammation and proliferation of CD^4+^ T cells could then spread towards extradigestive sites.

Since CD^4+^ T cells can cross the blood–brain barrier, they could give rise to central inflammatory responses. In this regard, the extensive recruitment of pro-inflammatory molecules and activated CD^4+^ T cells from the periphery is a common feature of CNS neurodegenerative disorders [39]. Moreover, the shift in the differentiation and number of CD^4+^ T-derived cell subtypes to proinflammatory phenotypes, such as Th1 and Th17, as well as the inhibition of Treg and Th2 anti-inflammatory activity, have been highlighted as important processes under such pathological conditions [40] (Figure 2). This pathological activation of CD^4+^ T cells has been associated with the induction of neuroinflammatory response and dysregulation, which represents a key event in the progressive development of neurodegenerative diseases, including PD [41,42] (Figure 2).

Overall, an important challenge for future work will be to evaluate how to reduce the detrimental consequences of intestinal reactive gliosis without compromising the physiological functions of EGCs. This can be daunting, considering that glial mediators, such as adenosine triphosphate (ATP) and NO, play essential roles in both GI physiology [19,43] and pathophysiology [44,45]. Therefore, targeting of the different signaling pathways involved in the physiological or pathological activities of the glia might allow the downregulation of pathological responses without interfering with GI physiology.

## 5. The Crosstalk between EGCs, Intestinal Epithelial Barrier and Gut Microbiota

**Figure 3:**
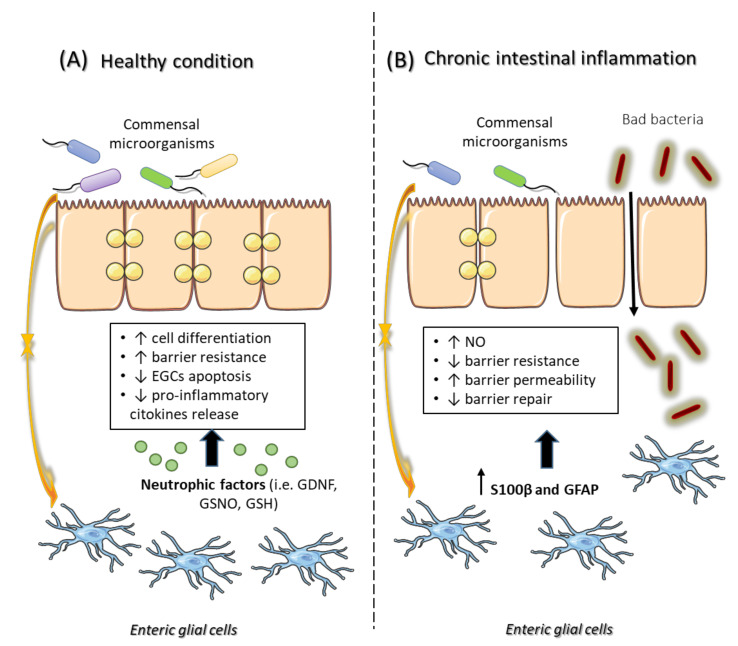
Role of EGCs in the regulation of intestinal epithelial barrier integrity under physiological and pathological conditions. (**A**) Under healthy conditions, the bidirectional interaction between gut microbiota and EGCs ensures the maintenance of GI homeostasis. In this context, EGCs play a pivotal role in the modulation of intestinal epithelial barrier homeostasis, regulating the IEC differentiation, proliferation, and apoptosis, and modulating the release of pro-inflammatory cytokines from IECs, through a wide range of glial mediators (i.e., GDNF and GSNO). (**B**) Changes in gut microbiota composition and a leaky intestinal epithelial barrier promote the translocation of bacteria or LPS from the intestinal lumen to the inner layer, triggering a pro-inflammatory response. Released cytokines and LPS then induce an increase in S100β/GFAP-positive EGCs, which secrete high amounts of NO with consequent further disruption of the intestinal epithelial barrier. Abbreviations: EGCs, enteric glial cells; GDNF, glial cell-derived neurotrophic factor; GFAP, glial fibrillary acidic protein; GI, gastrointestinal; GSNO, glial-derived S-nitrosoglutathione; IEC, intestinal epithelial cell; LPS, lipopolysaccharide; NO, nitric oxide.

The intestinal epithelium forms a controlled barrier that keeps the host separate from the contents of the bowel lumen, thus avoiding the passage of noxious substances, while allowing the absorption of nutrients [46]. An increasing body of evidence strongly supports the concept that the enteric glia is an effective regulator of physiological processes in the intestinal mucosa [47]. Indeed, molecular factors secreted by mucosal EGCs seem to be involved in the differentiation of epithelial cells and regulation of IEB homeostasis [48] (Figure 3).

For instance, mice with targeted enteric glia ablation display a drastic alteration of the epithelial barrier integrity [49]. In vitro studies have identified a variety of enteric glial-derived molecular factors that influence the activity of the gut mucosal barrier through specific actions on epithelial cells [50,51]. Among the different EGC mediators, GDNF seems to play a central role in the maintenance of mucosal integrity. Its protective activity is explicated by both inhibiting EGC apoptosis and reducing the release of pro-inflammatory cytokines [24,52] (Figure 3). Notably, in patients with functional dyspepsia, GDNF seems to be able to sustain the reconstitution and maturation of the epithelial barrier during moderate inflammation [53]. This action is closely related with the observation that EGCs display structural modifications in duodenal biopsies of patients with dyspepsia, characterized by overexpression of the protein S100B [54]. GDNF levels are increased in models of in vivo intestinal ischemia-reperfusion, perhaps as a defense mechanism. Indeed, under conditions of hypoxia/reoxygenation, GDNF was able to prevent epithelial barrier disruption [55]. Several studies indicate that EGC-derived GDNF improved the tight junctions in intestinal epithelial cells [56,57] (Figure 3). However, it is worth noting that EGCs are not the primary source of GDNF, as neurons and epithelial cells also produce and release this mediator [56].

Studies of human biopsies and primary cultured EGCs have shown that these cells express iNOS, thus having the ability of producing and releasing NO [58,59]. In human EGCs, this capability is enhanced by pro-inflammatory stimuli, such as lipopolysaccharide (LPS) [58], and pathogens [60] (Figure 3). Notably, S100B appears to exert a prominent role during EGC activation, and its overexpression and release has been correlated with NO hyperproduction during bowel inflammation [58,61]. Some studies have shown that a large proportion of NO release in gut inflammation may come from EGCs, and that EGC-derived NO correlates specifically with the integrity of the epithelial barrier [61,62] (Figure 3). Indeed, in an in vitro model of co-culture of EGCs with epithelial cells, it has been shown that, in the presence of LPS, glial NO acts on intestinal epithelial cell monolayers, increasing their permeability [63]. In addition, MacEachern et al. found that in a mouse model of colitis, EGC activation influences the mucosal transport of electrogenic ions, which results in an impaired IEB function, and that EGC-derived iNOS is directly responsible for this action (Figure 3). Likewise, the inhibition of EGCs with fluoroacetate, a glial cell metabolic toxin, and the prevention of NO production were able to restore the enteric barrier function in colitis [44].

Recently, unexpected findings have been made in contrast with the current theory that EGCs exert protecting actions on the enteric barrier [64]. For instance, under physiological conditions, the blockade of glial functions with fluoroacetate does not seem to affect the barrier functions [44]. Rao et al. observed that selectively ablating PLP-1 expressing glial cells did not trigger IEB disruption and epithelial alterations, and suggested that the mechanism by which the enteric glia compromises the IEB involves enteric inflammation and not the glial disruption itself [64]. Moreover, Grubišić and Gulbransen found that Cx43 ablation in enteric glia did not modulate the IEB functions [65]. However, it should be noted that many of these conflicting findings might be explained in light of the different approaches used to generate the results, (i.e., in vitro or in vivo), and the different methods employed to ablate EGCs.

One very interesting feature of EGCs is their ability to establish bidirectional interactions with the gut microbiota in the intestinal mucosa [66] (Figure 3). At present, it is not clear how EGCs specifically affect the microbiota. However, recent evidence suggests that the gut bacteria have a significant impact on mucosal glial growth. Kabouridis et al. [67] showed that the mucosal glia is continuously renewed by precursor cells and that such turnover does not take place in germ-free or antibiotic-treated mice. These findings suggest that stimuli from gut microbiota are crucial for facilitating glia migration from the enteric plexuses to the mucosa. During this process, even though the precise mechanisms remain still to be clarified, interactions of EGCs with both the microbiota and the immune system are required [67]. Hypothetically, it is possible that bacterial and viral components can affect actively the enteric glia through direct interactions with glial TLRs [68] (Figure 3). This would imply that glia is frequently exposed to bacterial/viral products in the mucosa, but it remains unknown to what extent these interactions occur under physiological conditions. Perhaps, a more plausible explanation is that the glia can be partially affected by the microbiota via interactions with the intestinal epithelium [69] and mucosal immune cells [70]. In this regard, neuroimmune interactions regulated by the microbiota have been reported already [71]. In this specific situation, through breaching the damaged epithelial layer, the luminal microbiota may act as a catalyst for neuroinflammation within the ENS. Remarkably, changes in intestinal microbiota composition and IEB disruption are closely correlated, and have been identified as main factors not only in bowel inflammation, but also in the pathogenesis of PD (Figure 3). This strong relationship is consistent with findings of altered gut microbiota composition in PD patients [6] and the occurrence of motor disorders in α-synuclein (α-syn)-overexpressing mice colonized with microbiota taken from PD patients [72]. Keshavarzian et al. [6] found that “anti-inflammatory” short chain fatty acid-producing bacteria, such as Blautia, Coprococcus, and Roseburia, were considerably reduced in the stool of PD patients, as well as Faecalibacterium in the mucosa, while “proinflammatory” Ralstonia were considerably more common in their bowel mucosa. In addition, about 25% of PD patients display an abnormal growth of small intestinal bacteria, which has been correlated directly to PD development [6]. While specific bacterial species have not yet been implicated in the development of PD, it has been reported that modifications of the intestinal microbiota can trigger an imbalance of the gut–brain axis through immunological priming of EGCs [73]. In support of these assumptions, rotenone was found to be partly effective in inducing PD in TLR-4 knockout mice, thus suggesting that an attenuated PD symptomatology depends on an impairment of EGC-mediated immune response [74].

Having an overall look at this body of knowledge, it is fair to conclude that a number of pathological intestinal conditions, associated with an impairment of barrier permeability, may trigger dysfunctions of EGCs and their shift towards a proinflammatory phenotype. Therefore, ameliorating the efficiency of mucosal barrier, as well as avoiding IEB disruption and the related reactive gliosis, might theoretically prevent the onset of PD or, at least, counteract its progression. However, for the time being, these are very thrilling observations that raise many questions about the microbiota–glial dynamics in gut physiology and pathophysiology.

## 6. Enteric α-Synuclein Accumulation in PD

In the CNS, the distinctive pathological feature of PD is the presence of insoluble deposits of misfolded α-syn in the basal ganglia, specifically in the substantia nigra. These protein deposits are commonly designated as Lewy bodies [75]. Interestingly, various clinical reports have revealed the presence of Lewy bodies and neurites also in the ENS of almost every investigated PD patient [76,77]. Several studies have shown that α-syn accumulation occurs in the ENS over the course of the pre-motor phase of the disease, thus greatly emphasizing the putative extra-CNS roots of this neurodegenerative disorder, and arguing strongly against the idea of PD as a central illness [78,79].

**Figure 4:**
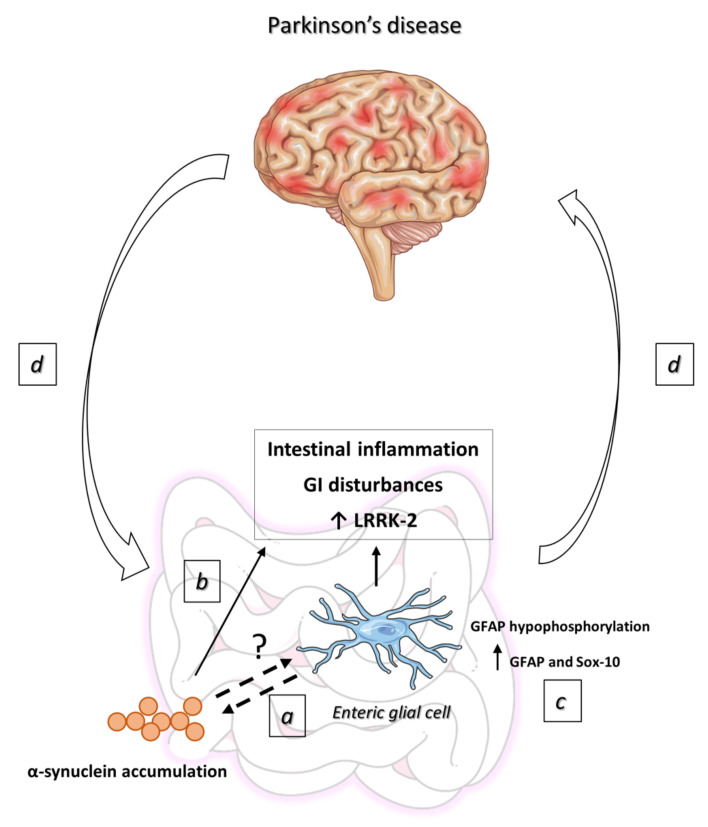
Schematic representation of the role of EGCs in the pathophysiology of PD. (**a**) Enteric α-synuclein accumulation in concomitance with alterations of EGC functions are distinctive pathological features of PD. In this setting, a “pathological loop” between glial activation by the aggregated α-synuclein and the induction of α-synuclein misfolding by the activated enteric glia has been reported, highlighting the involvement of EGCs in the etiology of PD. (**b**) The presence of misfolded α-synuclein is associated with the occurrence of GI disturbances and an increased expression of proteins linked to PD, such as LRRK-2. (**c**) The increase in Soxo-10/GFAP-positive EGCs, besides triggering intestinal motor dysfunctions, contributes to shape enteric immune/inflammatory responses. (**d**) Enteric immune/inflammatory events could trigger neuroinflammation and subsequent neurodegeneration in the CNS through gut–brain ascending pathways. In this setting, central neuroinflammation could, in turn, contribute to exacerbate enteric neuro-inflammatory conditions, through brain–gut descending pathways, triggering a sort of positive loop that could drive the chronicization of the ongoing inflammatory process. Abbreviations: CNS, central nervous system; EGCs, enteric glial cells; GFAP, glial fibrillary acidic protein; GI, gastrointestinal; LRRK-2, leucine-rich repeat kinase 2; PD, Parkinson’s disease.

In animal models, the presence of misfolded α-syn in the ENS is associated with the occurrence of GI disturbances and an increased expression of proteins linked to PD, such as leucine-rich repeat kinase 2 (LRRK-2) [80] (Figure 4).

A recent study showed that animals with experimental PD induced by systemic administration of LPS displayed an impairment of intestinal permeability in the initial stages of the disorder, prior to α-syn deposition in the ENS and the onset of central nigrostriatal neurodegeneration [81]. Moreover, Yang et al. [82] found the presence of intestinal dysbiosis, characterized by a rise in the ratio of Firmicutes/Bacteroidetes, enteric α-syn aggregation, and colonic inflammation in rodents with rotenone-induced PD. Of note, such alterations occurred prior to the onset of motor dysfunctions, central neurodegeneration, and appearance of α-syn inclusions in the CNS. In this setting, the alterations of the enteric bacteria-immune network might lead to a derangement of the gut–brain axis, resulting in a prionic migration of misfolded α-syn from the enteric compartment to CNS [83]. This suggest that, perhaps, the ENS could act as an entrance doorway to pathogens, contributing to the digestive pathogenesis of PD. In this regard, the vagus nerve appears to be involved in this process. Indeed, it has been observed that the effect of rotenone on PD-like development was inhibited by hemivagotomy [84]. Moreover, in a recent paper, Kim and colleagues demonstrated that α-syn fibrils, injected in the duodenal and pyloric muscular layer, can spread to the CNS, while a truncal vagotomy prevented the central α-synucleinopathy [85]. Additionally, it has been observed that patients with a truncal vagotomy had a lower risk of developing PD [86,87].

The typical motor manifestations of PD occur as a consequence of the loss of dopaminergic neurons in the substantia nigra, associated with the formation of Lewy bodies. In this regard, a large body of research has indicated also the occurrence of an early onset of Lewy pathology, in tandem with enteric glial dysfunction, in the colon of PD patients [88]. Therefore, the alterations of glial functions, in concomitance with an early accumulation of misfolded α-syn, may lead to changes in enteric neuronal activity and induction of intestinal inflammation, as well as dysfunctions of bowel motility, typically observed in PD patients (Figure 4). These concepts are in keeping with the clinical observation that the bowel and non-motor signs of PD appear many years earlier than the onset of motor impairment [89]. However, the mechanisms underlying the crosstalk between enteric α-syn and glial cells have not been clearly established and need to be investigated further.

## 7. EGCs in the Pathophysiology of PD

The increasing awareness of the essential role of EGCs in the regulation of GI physiology prompted the researchers to assess the putative impairments of this cell population in PD. A first important step has been to evaluate variations of the expression rates of the three widely known enteric glial markers, GFAP, S100β, and Sox-10. In this regard, Devos and colleagues first demonstrated that the expression levels of GFAP and Sox-10, but not S100β, were increased in colonic biopsies from PD patients [90]. Moreover, the expression of GFAP and Sox-10 were closely associated with increased levels of several pro-inflammatory cytokines, including IL-6 [36,90] (Figure 4).

GFAP is a phosphoprotein that needs to be phosphorylated in its amino-terminus to regulate its self-assembly, and plays a major role in cytoskeleton formation [91]. Given that cytoskeleton stabilization is important for normal astrocyte activity, alterations in the phosphorylation of GFAP might be critically involved in CNS disorders. Consistently with this hypothesis, alterations of central GFAP phosphorylation have been documented in both preclinical and clinical studies. Indeed, an increase in the phosphorylation of serine 13 residue has been reported both in a pig model of brain hypoxia [92] and the brain of patients with Alzheimer’s disease and frontotemporal dementia [93,94]. On the other hand, there is no evidence of altered GFAP phosphorylation in PD, and this point needs to be investigated.

On these bases, despite the lack of evidence in PD, it is conceivable that the occurrence of an impairment of GFAP phosphorylation might take place also in the GI tract. Consistently with this hypothesis, Clairembault et al. reported a hypophosphorylation in the serine 13 of GFAP in colonic biopsies from PD patients [95]. Intriguingly, these glial modifications appear to be unique to PD, since no changes in the expression of GFAP phosphorylation were found when examining colonic biopsies from patients with other neurodegenerative disorders [95]. Based on these findings, the different pattern of glial response in PD could probably depend on the different pathophysiological features of PD, as compared with other neurodegenerative disorders (Figure 4). Nevertheless, these observations suggest that reactive gliosis is strongly predictive of the GFAP changes found in the colon of PD patients [96]. Overall, these findings are consistent with the view that PD is not limited to the CNS, but is actually a generalized neuronal disease that affects peripheral autonomic networks, particularly the ENS, thus strengthening the theory that during PD, a glial reaction occurs in the GI tract [73]. In addition, the increase in enteric glial-related pro-inflammatory markers in the colon of PD patients, during the earliest stages of the disease, strongly suggests that an initial glial response takes place at the onset of this pathological condition [73] (Figure 4). Of note, glial marker levels are inversely proportional to the course of the disease, thus indicating that enteric glial activity is elevated at the beginning, while declining over time [90]. In particular, it is likely that enteric glial activity can be strong at the disease initiation, when triggered cells release their cytokines, while declining over the late phases of the disease, as observed previously with microglial activation in the brain [97].

As far as the involvement of EGCs in central neurodegeneration is concerned, early reactive enteric gliosis might promote a local neuroinflammation that could then ascend to CNS through glial Cx43 hemichannels, and likely via vagal nerve fibers. Glial Cx43 expression in the gap junctions depends on the concentration of intracellular Ca^2+^ and is strongly regulated by inflammation. For example, in the HIV-1 Tat-induced diarrhea model, an overexpression of Cx43 in S100B-positive cells has been shown, with a propagation of this expression pattern from the intestinal submucosal plexus to the frontal cortex, through the spinal cord, suggesting that the connection between EGCs and central astrocytes involves such glial proteins [61]. In this model, a late onset of cognitive function disorder was observed as a result of such mechanisms [61].

The gut–brain axis is a bidirectional signaling pathway, through which, on the one hand, the gut is able to interact with the brain, and on the other hand, CNS alterations and neurodegeneration may affect the GI tract (Figure 4). In a recent paper, Pellegrini and colleagues observed that the induction of central nigrostriatal neurodegeneration with 6-hydroxydopamine (6-OHDA) in rats is followed by major changes in colonic excitatory neuromotility [98]. Moreover, they found evident signs of colonic inflammation in tissues from 6-OHDA rats, characterized by elevated levels of TNF, IL-1β, and oxidative stress [98]. Of note, these results are consistent with previous clinical findings that showed a rise in pro-inflammatory cytokine levels, including TNF and IL-1β, and signs of glial cell activation (i.e., an increase in ganglionic GFAP levels) in colonic biopsies from patients with PD [90].

Furthermore, colonic tissues from 6-OHDA rats displayed significant histopathological changes ranging from mucosal barrier alterations with leukocyte infiltration, decrease in epithelial claudin, and increase in S100-positive mucosal glia, up to a decrease in interstitial cells of Cajal, the pacemakers and mediators of gut neurotransmission [99].

Even if some of the mechanisms underlying the role of EGCs in the gut–brain communication have been clarified, much more still remain to be elucidated. Thus, further investigations are needed to better understand the origins of reactive gliosis, which could be triggered by changes in peripheral tissues (i.e., dysbiosis or external noxae) or descending stimuli from CNS.

## 8. Conclusions

Current knowledge supports the view that the GI dysfunctions and glial changes found in PD patients are critical events for the initiation of neurodegeneration and are closely related with the disruption of IEB and alterations of gut microbiota composition [100]. In this context, an abnormal activation of EGCs is likely responsible for neuroinflammation and the associated pathological changes in the ENS. IEB impairment, along with the involvement of EGC-mediated neuroinflammation in the early stages of PD development [88], makes PD a multicentric disease and emphasizes the role of EGCs in the initiation and spread of neurodegenerative processes to the CNS.

Glial involvement in the development of PD is consistent with Braak’s studies, which showed that α-syn misfolding tends to occur first in the peripheral autonomic nervous system, particularly in the axon terminals of submucosal plexus and neurons of the myenteric plexus [101]. Afterwards, misfolded α-syn would migrate to the nigrostriatal areas of CNS in a prion-like way. A diffusion of α-syn pathology in a “prion-like” fashion via a pathway involving glia to glia interactions has been postulated. However, the precise mechanisms accounting for the transmission of PD pathology to the nigrostriatal region remain unknown.

While more studies are needed to validate the exact role of EGCs in the onset of PD and its progression from the intestine to CNS, it is conceivable that current knowledge might foster the identification of novel therapeutic interventions targeting the functions of EGCs in the early stages of the disease. In this regard, administering probiotics or eubiotics to modulate the dysbiosis, and developing medications able to reduce IEB impairment and EGC-induced neuroinflammation, might theoretically prevent or counteract the neurodegeneration ascending towards the CNS.
